# Overall and cause-specific mortality among patients diagnosed with gastric precancerous lesions in Sweden between 1979 and 2014: an observational cohort study

**DOI:** 10.1186/s12916-024-03554-1

**Published:** 2024-08-15

**Authors:** Yawen Sun, Li Yin, Dariush Nasrollahzadeh Nesheli, Jingru Yu, Joar Franzén, Weimin Ye

**Affiliations:** 1 Shandong Cancer Hospital and Institute, Shandong First Medical University and Shandong Academy of Medical Sciences, Jinan, Shandong 250117 China; 2 Department of Medical Epidemiology and Biostatistics, Karolinska Institutet, Stockholm, 17177 Sweden

**Keywords:** Gastric precancerous lesions, Overall mortality, Cause-specific mortality, Cancer, Non-cancer conditions

## Abstract

**Background:**

The Correa’s cascade, encompassing chronic non-atrophic gastritis, atrophic gastritis, intestinal metaplasia, and dysplasia, represents the well-recognized pathway for the development of non-cardia gastric cancer. Population-based studies on all-cause and cause-specific mortalities among patients with gastric lesions in Correa’s cascade are scarce.

**Methods:**

We compiled a cohort of 340 744 eligible patients who had undergone endoscopy with biopsy for non-malignant indications during the period 1979–2011, which was followed up until 2014. Standardized mortality ratios (SMRs) with 95% confidence intervals (CIs) provided estimation of the relative risk, using the general Swedish population as reference. Cox regression model was used to estimate hazard ratios (HRs) of death for internal comparison.

**Results:**

A total of 306 117 patients were included in the final analysis, accumulating 3,049,009 person-years of follow-up. In total 106,625 deaths were observed during the study period. Compared to the general population, excess risks of overall mortality were noted in all subgroups, with SMRs ranging from 1.11 (95% CI 1.08–1.14) for the normal mucosa group to 1.54 (95% CI 1.46–1.62) for the dysplasia group. For cause-specific mortalities, mortality from gastric cancer gradually increased along Correa’s cascade, with excess risk rising from 105% for patients with chronic gastritis to more than 600% for the dysplasia group. These results were confirmed in the comparison with the normal mucosa group. For non-cancer conditions, increased death risks were noted for various diseases compared to the general population, especially among patients with more severe gastric precancerous lesions. But the results were confirmed only for “infectious diseases and parasitic diseases”, “respiratory system diseases”, and “digestive system disease”, when using the normal mucosa group as reference.

**Conclusions:**

Increased mortality from gastric cancer suggests that early recognition and intervention of gastric precancerous lesions probably benefit the patients. Excess mortality due to non-cancer conditions should be interpreted with caution, and future studies are warranted.

**Supplementary Information:**

The online version contains supplementary material available at 10.1186/s12916-024-03554-1.

## Background

The Correa’s cascade, comprising a sequential progression from chronic non-atrophic gastritis to atrophic gastritis, intestinal metaplasia, and dysplasia, is a well-recognized pathway for the development of non-cardia gastric cancer [1]. Some patients with these lesions will eventually develop invasive gastric adenocarcinoma if appropriate intervention or treatment is not taken [2, 3]. Globally, 1 in 12 cancer deaths is caused by gastric cancer, making it the third leading cause of cancer death [4]. And the prognosis of gastric cancer patients remains dismal. In the USA, no significant improvement in survival was reported for patients with non-cardia gastric adenocarcinoma during the past 20 years [5]. The distribution of tumor stage at gastric cancer diagnosis and 5-year survival both worsened over time among patients in the Netherlands [6].

Our earlier study examined stomach cancer incidence among patients with different mucosal lesions in Sweden. The results indicated patients with lesions along Correa’s cascade had an increasingly higher incidence of gastric cancer in comparison to that of the general population [7]. In 2020, Boreiri et al. conducted a prospective study, involving endoscopic examination and biopsy on 1011 residents in Adabir, Iran, and revealed that gastric precancerous lesions, including atrophic gastritis and intestinal metaplasia, significantly increased the risk of death from gastric cancer [8]. However, a comprehensive analysis of mortality and causes of death among patients with gastric precancerous lesions remains elusive. Arguably, the most significant indicator of health inequality for any population group is excess mortality relative to the general population. An understanding of the drivers of excess mortality is important for patients with gastric precancerous lesions and for those concerned with their lifetime needs, such as family members, health professionals, and policymakers.

Starting from the 1970s, pathology departments in Sweden have been recording the histopathological results for patients undergoing gastroscopy and with tissue biopsy. Taking advantage of these completely computerized registrations, we launched a large-scale, long-time observational cohort study, to describe the overall and cause-specific mortality profiles for patients diagnosed with gastric precancerous lesions at baseline. For these patients, we further quantified their excess risks of death relative to the entire Swedish population and patients with normal gastric mucosa.

## Methods

### Study design

From 1979 to 1998, 24 pathology departments in Sweden built up computerized registers gradually [7]. The collected data included patients’ national registration numbers (unique identifiers for all Swedish residents) along with other variables such as date, age, sex, and pathological-anatomical diagnosis utilizing the Systematized Nomenclature of Medicine Morphology (SNOMED M) codes. Based on these registers, we created a cohort enrolling all patients who underwent gastroscopic biopsy for non-malignant indications. Up to Dec 31, 2014, we identified 445,664 subjects with at least one examination record. Causes and date of death were ascertained by cross-linkage to the Swedish Cause of Death Register. We also retrieved information from the Swedish Death Register for double check of death date and further cross-linked with the Swedish Emigration Register for censoring. Linkage to the Education Register provided information of the highest level of education achieved by the subjects.

**Fig. 1:**
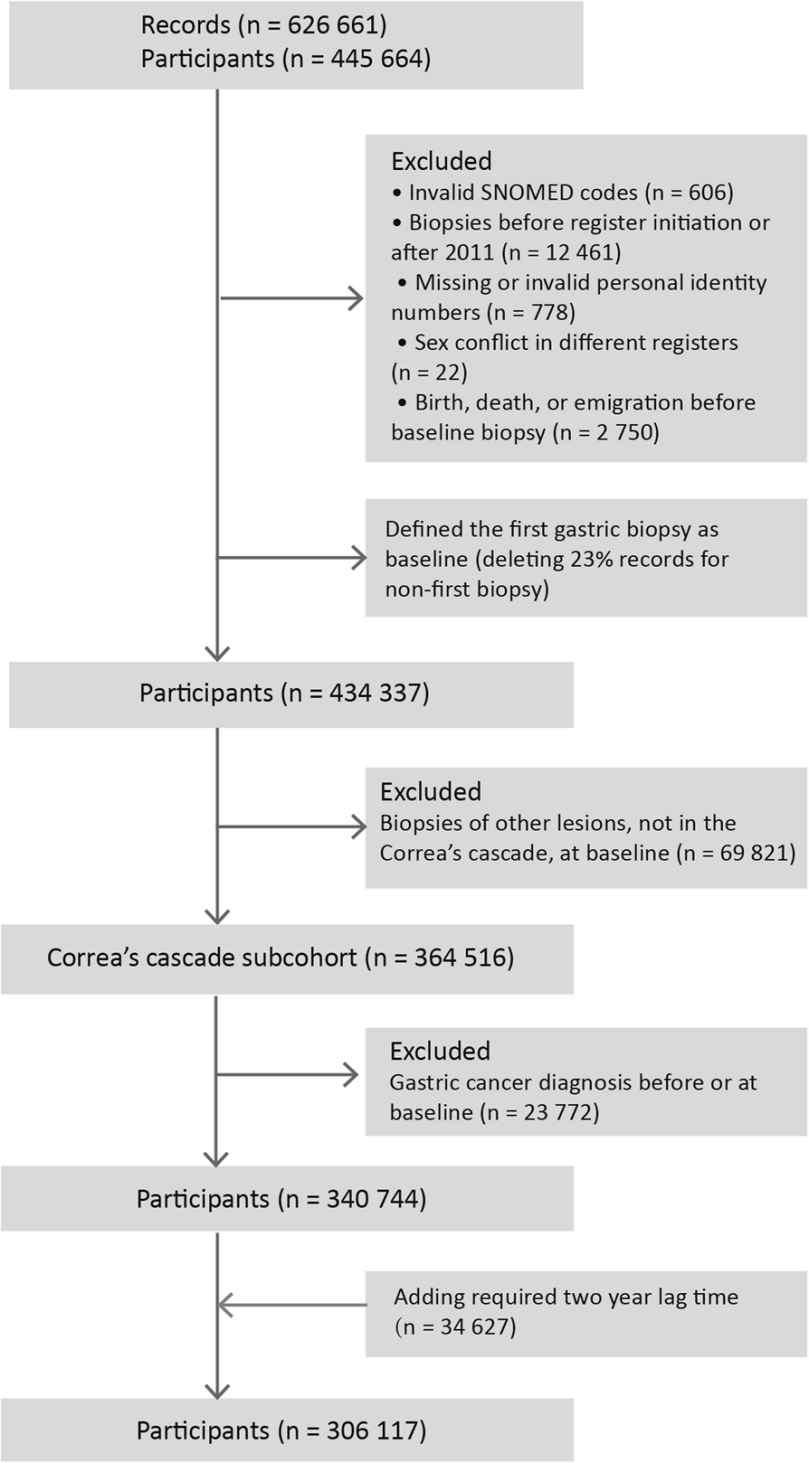
Study design for stomach biopsy cohort in Sweden (1979–2014). Baseline is defined as the first biopsy identified in the database. When multiple diagnoses were present, the most severe one was selected

As described previously, we limited subjects enrolled between 1979 and 2011, and after further exclusion, 434,337 participants remained in the cohort [9]. For each participant, the first biopsy date was defined as the date of enrollment in the database. We grouped the cohort members by their SNOMED M diagnosis at baseline (for detailed information please see Table S1). This study focused on gastric mucosal status within Correa’s cascade, categorized into normal, minor mucosal change, chronic gastritis, atrophic gastritis, intestinal metaplasia, and dysplasia. So, we further excluded patients not in Correa’s cascade at baseline (*n* = 69,821) and those with gastric cancer at or before baseline (*n* = 23,772). The study cohort finally included 340,744 eligible patients (Fig. 1). We continued follow-up until death, emigration, or the end of follow-up (Dec 31, 2014), whichever occurred first. We categorized deaths broadly as due to gastric cancer, non-gastric cancers, and non-cancer conditions. And non-cancer conditions were defined by organ system. The diagnosis of cause-specific death used the corresponding International Classification of Diseases (ICD) codes (Table S2).

### Statistical analysis

To measure the absolute risk of mortality, we calculated crude death rates for gastric cancer, non-gastric cancers, and non-cancer conditions by dividing the number of deaths with the observed person-years. We also calculated standardized mortality ratios (SMRs) by dividing the observed number by the expected number of deaths, derived by multiplying sex-, age- (in 5-year intervals), and calendar year-specific mortality rates for the general population with the person-years at risk accrued in our cohort. The 95% confidence intervals for the crude death rate and SMRs were calculated assuming Poisson distribution for the number of deaths.

We regularly observed a peak of mortality emerging hastily after the first gastric biopsy among the study population probably due to undetected cancer patients, indirectly resulting in an overestimation of the death risk for the study population. Therefore, to minimize the influence of selection bias, we chose to start follow-up when this peak had passed. Thus, in our main analysis, we discarded the first 2 years of observation and deaths detected during this period.

We used hazard ratios with 95% confidence intervals, derived from the Cox proportional hazards regression model, to evaluate the association between gastric mucosal status and risks of deaths.

The time scale used was the attained age. The regression model was adjusted for age, sex, and education level, as well as calendar year as a time-dependent covariate. The proportional hazards assumption was checked graphically and by Schoenfeld’s partial residuals; neither of them revealed any indication of violation of this assumption. *P* values less than 0.05 were considered to indicate statistical significance. The results were all analyzed using SAS statistical software, version 9.3 (Cary, NC).

## Results

After adding the required lag time of 2 years, we finally identified 306 117 patients whose diagnoses were classified into Correa’s cascade. Of the total patients, 44.58% were male. Mean (± SD) age at cohort entry was 56.64 ± 19.37 years. The mean duration of follow-up was 9.96 years, and 3,049,009 person-years at risk were accumulated (Table 1). Chronic gastritis comprised more than 58% of the entire cohort, contrasted by less than 1% for dysplasia. Patients with more advanced gastric precancerous lesions tended to be older than those with milder lesions. Except dysplasia group, males were less dominant in other subgroups. The normal mucosa (10.42 years) and chronic gastritis (10.38 years) group had the longest mean follow-up duration, while the intestinal metaplasia group had the shortest (6.99 years).

**Table 1:** Characteristics of patients enrolled in stomach biopsy cohort

Mucosal status at baseline^a^	No of participants (%)	Mean (SD) age at cohort entry (years)^b^	% male	Mean (SD) follow-up duration (years)^b^	Person-years^b^
Overall	306117 (100.00)	56.64 (19.37)	44.58	9.96 (6.53)	3049009
Normal	33052 (10.80)	46.40 (20.60)	41.18	10.42 (6.00)	344468
Minor mucosal changes	67111 (21.92)	50.03 (19.76)	40.89	9.20 (6.21)	617522
Chronic gastritis	177647 (58.03)	58.30 (18.18)	46.60	10.38 (6.74)	1843404
Atrophic gastritis	14981 (4.89)	60.25 (18.49)	41.36	9.69 (6.59)	145233
Intestinal metaplasia	11058 (3.61)	66.22 (14.78)	47.67	6.99 (4.61)	77338
Dysplasia	2268 (0.74)	65.52 (14.78)	51.94	9.28 (6.70)	21044

^a^Defined as the first biopsy identified in the database. When multiple diagnoses were present, the most severe one was selected

^b^Calculated after exclusion of first 2 years of follow-up

A total of 106 625 deaths occurred during follow-up. Table 2 presents the distribution of death cases along with crude mortality rate and SMR, from normal mucosa to dysplasia groups. For overall mortality, all subgroups showed an increased risk compared to the general population, with SMRs ranging from 1.11 for the normal mucosa group to 1.54 for the dysplasia group. For gastric cancer death, the mortality rate in the normal group was 16.26 × 10^−5^ (95% CI 12.51–21.13), without showing a statistically, significantly increased death risk (SMR 1.18, 95% CI 0.87–1.49) compared to the Swedish general population. Then, the mortality increased stepwise along Correa’s cascade. For patients with chronic gastritis, the mortality was 54.19 × 10^−5^ (95% CI 50.94–57.66), corresponding to a 105% excess risk (95% CI 92–118%). For the dysplasia group, the mortality increased to 237.60 × 10^−5^ (95% CI 180.08-313.49), which corresponded to a SMR of 7.01 (95% CI 5.06–8.95). For non-gastric cancers, modest excess mortalities were also evidenced among all subgroups, ranging from 8% (atrophic gastritis, 95% CI 1–14%) to 53% (dysplasia, 95% CI 35–72%). For non-cancer conditions, a conspicuously elevated risk of death from “infectious and parasitic disease” was observed among the patients with dysplasia (SMR 2.56, 95% CI 1.73–3.40), whereas moderately increased mortality risks varied from 26 to 48% in other groups. For “Endocrine, nutritional and metabolic disease”, excess mortality was from 30 to 81%, showing an irregular upward trend among subgroups, with dysplasia (SMR 1.81, 95% CI 1.23–2.39) having the highest excess death risk. The generally increased mortalities from “respiratory system disease” were observed in all subgroups, among which patients with dysplasia (SMR 1.91, 95% CI 1.57–2.25) had the most prominently increased risk of death. Compared with the Swedish population, patients with dysplasia (SMR 2.23, 95% CI of 1.65–2.81) had the highest risk of death from “digestive system diseases”, followed by patients with minor mucosal changes (SMR 1.93, 95% CI 1.79–2.08) and chronic gastritis (SMR 1.92, 95% CI 1.85–1.98).

**Table 2:** All-cause and cause-specific mortalities among subjects undergoing endoscopy, stratified by mucosal status at baseline^a^

	**Normal**			**Minor mucosal changes**			**Chronic gastritis**		
**Cause of death**	**No of death**^**b**^	**Crude mortality rate**^**c**^	**SMR (95% CI)**^**d**^	**No of death**^**b**^	**Crude mortality rate**^**c**^	**SMR (95% CI)**^**d**^	**No of death**^**b**^	**Crude mortality rate**^**c**^	**SMR (95% CI)**^**d**^
**All cause**	6190	1796.98	1.11 (1.08–1.14)	14628	2368.83	1.26 (1.24–1.28)	74044	4016.70	1.28 (1.27–1.29)
**Cancers**	1490	432.55	1.11 (1.05–1.17)	3512	568.73	1.28 (1.24–1.32)	15212	825.21	1.24 (1.22–1.26)
Gastric cancer	56	16.26	1.18 (0.87–1.49)	156	25.26	1.59 (1.34–1.84)	999	54.19	2.05 (1.92–2.18)
Non-gastric cancers	1434	416.29	1.11 (1.05–1.16)	3356	543.46	1.27 (1.23–1.31)	14213	771.02	1.21 (1.19–1.23)
**Non-cancer conditions**	4700	1364.42	1.11 (1.08–1.14)	11116	1800.10	1.25 (1.23–1.28)	58833	3191.54	1.29 (1.28–1.30)
Infectious and parasitic disease	121	35.13	1.26 (1.03–1.48)	299	48.42	1.48 (1.31–1.65)	1283	69.60	1.38 (1.30–1.45)
Endocrine, nutritional, and metabolic disease	178	51.67	1.30 (1.11–1.49)	422	68.34	1.48 (1.34–1.62)	1987	107.79	1.45 (1.39–1.52)
Mental and behavioral disorder	235	68.22	0.81 (0.71–0.91)	584	94.57	0.96 (0.88–1.04)	3170	171.97	1.05 (1.01–1.08)
Nervous system	198	57.48	1.09 (0.94–1.24)	444	71.90	1.18 (1.07–1.29)	1899	103.02	1.15 (1.09–1.20)
Circulatory system	2444	709.50	1.03 (0.99–1.07)	5899	955.27	1.18 (1.15–1.21)	33057	1793.26	1.23 (1.22–1.25)
Respiratory system	469	136.15	1.24 (1.13–1.35)	1069	173.11	1.35 (1.26–1.43)	5924	321.36	1.42 (1.38–1.45)
Digestive system	294	85.35	1.67 (1.48–1.86)	700	113.36	1.93 (1.79–2.08)	3353	181.89	1.92 (1.85–1.98)
Genitourinary system	90	26.13	1.22 (0.96–1.47)	253	40.97	1.61 (1.41–1.81)	1220	66.18	1.43 (1.35–1.51)
Symptoms, signs, and ill-defined conditions	155	45.00	0.94 (0.79–1.09)	345	55.87	1.01 (0.91–1.12)	1848	100.25	1.11 (1.06–1.16)
**Cause of death**	**Atrophic gastritis**			**Intestinal metaplasia**			**Dysplasia**		
	**No of death**^**b**^	**Crude mortality rate**^**c**^	**SMR (95% CI)**^**d**^	**No of death**^**b**^	**Crude mortality rate**^**c**^	**SMR (95% CI)**^**d**^	**No of death**^**b**^	**Crude mortality rate**^**c**^	**SMR (95% CI)**^**d**^
**All cause**	6197	4266.95	1.17 (1.14–1.20)	4240	5482.44	1.25 (1.21–1.29)	1326	6301.12	1.54 (1.46–1.62)
**Cancers**	1218	838.66	1.12 (1.06–1.19)	874	1130.11	1.28 (1.19–1.36)	314	1492.12	1.75 (1.56–1.94)
Gastric cancer	98	67.48	2.31 (1.85–2.77)	67	86.63	2.88 (2.19–3.57)	50	237.60	7.01 (5.06–8.95)
Non-gastric cancers	1120	771.18	1.08 (1.01–1.14)	807	1043.47	1.22 (1.14–1.31)	264	1254.52	1.53 (1.35–1.72)
**Non-cancer conditions**	4979	3428.30	1.18 (1.14–1.21)	3366	4352.33	1.24 (1.20–1.29)	1012	4809.00	1.48 (1.39–1.57)
Infectious and parasitic disease	112	77.12	1.27 (1.04–1.51)	90	116.37	1.35 (1.07–1.63)	36	171.07	2.56 (1.73–3.40)
Endocrine, nutritional, and metabolic disease	196	134.96	1.57 (1.35–1.78)	115	148.70	1.38 (1.12–1.63)	37	175.82	1.81 (1.23–2.39)
Mental and behavioral disorder	308	212.07	1.06 (0.94–1.17)	219	283.17	1.02 (0.88–1.15)	50	237.60	1.10 (0.80–1.41)
Nervous system	172	118.43	1.11 (0.95–1.28)	112	144.82	0.96 (0.78–1.14)	31	147.31	1.26 (0.82–1.71)
Circulatory system	2879	1982.34	1.16 (1.12–1.20)	1907	2465.80	1.25 (1.19–1.30)	547	2599.33	1.36 (1.25–1.47)
Respiratory system	464	319.49	1.20 (1.09–1.31)	353	456.44	1.48 (1.33–1.63)	121	574.99	1.91 (1.57–2.25)
Digestive system	202	139.09	1.28 (1.10–1.45)	152	196.54	1.53 (1.29–1.78)	57	270.86	2.23 (1.65–2.81)
Genitourinary system	103	70.92	1.29 (1.04–1.54)	63	81.46	1.24 (0.93–1.54)	22	104.54	1.66 (0.97–2.35)
Symptoms, signs, and ill-defined conditions	156	107.41	0.98 (0.83–1.14)	102	131.89	0.88 (0.71–1.05)	22	104.54	0.89 (0.52–1.26)

^a^Defined as first biopsy identified in database. When multiple diagnoses were present, the most severe one was selected

^b^First 2 years of observation and corresponding events were excluded

^c^Per 100,000 person-years

^d^Observed to expected number of death cases, based on age (5-year strata), sex, and calendar year (5-year strata) specific mortality rates in total Swedish population. Ninety-five percent confidence intervals of SMRs were calculated by assuming that observed death occurrence followed a Poisson distribution

To further explore the potential association between grade of lesion and death risk, we separately calculated hazard ratios (HRs) with 95% CIs across gastric precancerous lesion groups by cause of death, using the normal group as reference, to minimize the influence of selection bias (Table 3). For the all-cause death, there was a 1.09 (minor mucosal changes, 95% CI 1.06–1.13) to 1.33-fold (dysplasia, 95% CI 1.26–1.42) increased risk of death in patients with gastric precancerous lesions compared to the normal mucosa group, while HR for the atrophic gastritis group was non-significant (1.02, 95% CI 0.99–1.06). Gastric cancer death strongly correlated with having dysplasia, with a nearly sixfold increased death risk (HR 5.85, 95% CI 3.95–8.66). Less conspicuous excesses, but still substantial and statistically significant, were also seen in groups of intestinal metaplasia (HR 2.63, 95% CI 1.82–3.79), atrophic gastritis (HR 2.03, 95% CI 1.45–2.85), and chronic gastritis (HR 1.71, 95% CI 1.29–2.26). For non-gastric cancer deaths, the mortality risk of patients with minor mucosal changes (HR 1.13, 95% CI 1.06–1.21), chronic gastritis (HR 1.07, 95% CI 1.01–1.13), intestinal metaplasia (HR 1.14, 95% CI 1.05–1.25), and dysplasia (HR 1.34, 95% CI 1.18–1.54) was higher than that of the normal mucosa group. For non-cancer conditions, the death risk of “infectious diseases and parasitic diseases” was appreciably raised in the dysplasia group (HR 2.02, 95% CI 1.38–2.95). Most notably, the mortality risk of patients with chronic gastritis (HR 1.13, 95% CI 1.03–1.25), intestinal metaplasia (HR 1.23, 95% CI 1.06–1.42), and dysplasia (HR 1.48, 95% CI 1.20–1.81) were significantly elevated for “respiratory system disease” when they were compared to that of normal mucosa group. For death from “digestive system disease”, the HRs (95% CIs) were 0.80 (0.66–0.96) and 1.36 (1.02–1.81) for groups of atrophic gastritis and dysplasia, respectively, showing opposite trends in death risks. Remarkably, for “Endocrine, nutritional and metabolic disease”, HRs (95% CIs) across the 5 groups of gastric lesions were all without statistical significance.

**Table 3:** Hazard ratios (HRs) and 95% confidence intervals (CI) for overall and cause-specific deaths among patients with different gastric lesions compared to the normal mucosal group

**Cause of death**	**Normal**	**Minor mucosal changes**			**Chronic gastritis**			**Atrophic gastritis **		
		**No of death**^**a**^	**HRs (95% CI)**^**b**^	***P* value**	**No of death**^**a**^	**HRs (95% CI)**^**b**^	***P* value**	**No of death**^**a**^	**HRs (95% CI)**^**b**^	***P* value**
**All cause**	Reference	14628	1.09 (1.06–1.13)	< 0.0001	74044	1.11 (1.08–1.15)	< 0.0001	6197	1.02 (0.99–1.06)	0.2201
**Cancers**	Reference	3512	1.13 (1.06–1.21)	0.0001	15212	1.09 (1.03–1.16)	0.0021	1218	1.01 (0.94–1.09)	0.7645
Gastric cancer	Reference	156	1.23 (0.89–1.69)	0.2037	999	1.71 (1.29–2.26)	0.0002	98	2.03 (1.45–2.85)	< 0.0001
Non-gastric cancers	Reference	3356	1.13 (1.06–1.21)	0.0003	14213	1.07 (1.01–1.13)	0.0250	1120	0.97 (0.90–1.05)	0.4770
**Non-cancer conditions**	Reference	11116	1.08 (1.04–1.12)	< 0.0001	58833	1.12 (1.09–1.16)	< 0.0001	4979	1.03 (0.99–1.07)	0.2203
Infectious and parasitic disease	Reference	299	1.12 (0.90–1.40)	0.3228	1283	1.05 (0.87–1.28)	0.6059	112	0.99 (0.76–1.29)	0.9291
Endocrine, nutritional, and metabolic disease	Reference	422	1.02 (0.89–1.23)	0.8162	1987	1.09 (0.92–1.28)	0.3170	196	1.20 (0.97–1.48)	0.0984
Mental and behavioral disorder	Reference	584	1.14 (0.97–1.34)	0.1104	3170	1.21 (1.06–1.40)	0.0064	308	1.19 (1.00–1.41)	0.0554
Nervous system	Reference	444	1.02 (0.85–1.21)	0.8558	1899	1.01 (0.86–1.18)	0.9169	172	0.95 (0.77–1.18)	0.6554
Circulatory system	Reference	5899	1.07 (1.02–1.12)	0.0091	33057	1.12 (1.07–1.17)	< 0.0001	2879	1.05 (0.99–1.11	0.1200
Respiratory system	Reference	1069	1.08 (0.97–1.22)	0.1677	5924	1.13 (1.03–1.25)	0.0131	464	0.97 (0.85–1.10)	0.6225
Digestive system	Reference	700	1.15 (1.00–1.33)	0.0638	3353	1.16 (1.03–1.32)	0.0185	202	0.80 (0.66–0.96)	0.0163
Genitourinary system	Reference	253	1.30 (1.01–1.67)	0.0441	1220	1.15 (0.92–1.44)	0.2076	103	1.05 (0.78–1.40)	0.7607
Symptoms, signs, and ill-defined conditions	Reference	345	1.23 (1.01–1.50)	0.0413	1848	1.30 (1.10–1.55)	0.0026	156	1.21 (0.96–1.52)	0.1065
**Cause of death**	**Normal**	**Intestinal metaplasia**			**Dysplasia**					
		**No of death**^**a**^	**HRs (95% CI)**^**b**^	***P* value**	**No of death**^**a**^	**HRs (95% CI)**^**b**^	***P* value**			
**All cause**	Reference	4240	1.15 (1.10–1.19)	< 0.0001	1326	1.33 (1.26–1.42)	< 0.0001			
**Cancers**	Reference	874	1.20 (1.19–1.31)	< 0.0001	314	1.58 (1.35–1.73)	< 0.0001			
Gastric cancer	Reference	67	2.63 (1.82–3.79)	< 0.0001	50	5.85 (3.95–8.66)	< 0.0001			
Non-gastric cancers	Reference	807	1.14 (1.05–1.25)	0.0031	264	1.34 (1.18–1.54)	< 0.0001			
**Non-cancer conditions**	Reference	3366	1.13 (1.08–1.19)	< 0.0001	1012	1.28 (1.20–1.37)	< 0.0001			
Infectious and parasitic disease	Reference	90	1.13 (0.85–1.50)	0.4056	36	2.02 (1.38–2.95)	0.0003			
Endocrine, nutritional, and metabolic disease	Reference	115	1.11 (0.87–1.42)	0.3868	37	1.37 (0.95–1.96)	0.0900			
Mental and behavioral disorder	Reference	219	1.22 (1.01–1.48)	0.0387	50	1.24 (0.91–1.69)	0.1676			
Nervous system	Reference	112	0.88 (0.70–1.12)	0.3146	31	1.18 (0.81–1.74)	0.3932			
Circulatory system	Reference	1907	1.15 (1.08–1.23)	< 0.0001	547	1.23 (1.12–1.35)	< 0.0001			
Respiratory system	Reference	353	1.23 (1.06–1.42)	0.0053	121	1.48 (1.20–1.81)	0.0002			
Digestive system	Reference	152	1.05 (0.86–1.28)	0.6536	57	1.36 (1.02–1.81)	0.0383			
Genitourinary system	Reference	63	1.03 (0.74–1.43)	0.8695	22	1.34 (0.83–2.15)	0.2291			
Symptoms, signs, and ill-defined conditions	Reference	102	1.14 (0.88–1.48)	0.3225	22	1.01 (0.64–1.58)	0.9817			

^a^First 2 years of observation and corresponding events were excluded

^b^Using attained age as an underlying time scale, estimated by Cox proportional hazards regression model, adjusted for sex, education status, and calendar year as a time-dependent covariate, and stratified by the pathology department

## Discussion

In this nationwide pathology-based cohort study, we used two complementary measures to explore the association between abnormal gastric mucosal histology and all causes of death among the Swedish population. We found an excess mortality rate due to diseases involving organs and systems boundless of the gastrointestinal system (GI) associated with an abnormal stomach biopsy 2 years after a gastroscopy.

An attractive feature of cohort studies is the capability they provide to examine a range of health outcomes such as follow-up for mortality from different causes of death. The cohort in this study is not defined by population or health condition. Alternatively, an index event of histology reporting of gastric biopsy defines the current cohort. In this cohort, having a stomach histology report is the index health event, not the health indication for which esophago-gastro-duodenoscopy (EGD) was prescribed. Under this setting, those who underwent EGD without performing a gastric biopsy would not be included. Also, this study is blind toward the comorbidities in the esophagus or duodenum and their possible pathology reports during the same EGD session when gastric biopsy was taken. Likewise, multimorbidity in other organs was not considered, which may potentially introduce some selection bias toward diseases or treatments which cause gastric mucosal inflammation and anemia such as cardiovascular diseases and accompanying NSAID or anticoagulant administration [10]. Mortality due to diagnostic or therapeutic EGD complications has no effect on final results because the first 2-year mortality was not included in the analysis.

People with a family history of gastric diseases and cancer may seek or be referred more often to an endoscopy unit. In this cohort, a portion of the excess mortality rate might not be a biological effect of abnormal gastric mucosa but would instead be an effect of personal concern on the detection of gastrointestinal problems.

By selecting the most advanced stage based on Correa’s model as an index event at the start of the follow-up, we fixed the cohort by blocking the possibility of movement of individuals between chronic exposure groups during the follow-up. Hypothetically, the early steps of Correa’s model are reversible upon efficient *H. pylori* eradication. Clinically, active chronic gastritis is considered a lifelong condition without treatment. Prospective studies have shown that low-grade dysplasia may regress up to 60% of cases, whereas 10–20% progress to high-grade dysplasia [11]. In our sensitivity test among 21% of repeated EGD, progression and regression in gastric mucosal status were not pronounced for two ends of the range (normal and dysplasia) [7]. Collectively, at worst, this study design would underestimate the effect size of dysplasia on the mortality rate. In contrast, due to probable exposure dynamics, our analysis may overestimate the effect size of minor mucosal changes on the outcomes.

Exposure in this cohort was defined based on the sequence of gastritis, atrophy, intestinal metaplasia, and dysplasia which was first popularized by Correa as a progressive model for gastric adenocarcinoma development. *H. pylori* is the most common cause of chronic gastritis and initiation of Correa’s cascade. Since microscopy is the least reliable method for detecting *H. pylori* infection, accurate data on *H. pylori* status was unavailable. As a result, it is probable that a proportion of individuals categorized as normal mucosa group would be *H. pylori* positive. Of note, non-*H. pylori* chronic gastritis also occurs not infrequently and is associated with proton pump inhibitors (PPI) use. This condition may dilute the effect size of the observed association between gastric mucosal status and mortality when the normal mucosa group was considered as a reference in the analysis. Another indirect evidence for the possibility of admixture of the normal group with *H. pylori*-positive or other esophageal or duodenal comorbidities is the higher risk of mortality due to digestive diseases among the normal mucosa group in this cohort in comparison with the general population.

Generally, histology does not yield equal precision for the diagnosis of gastritis, atrophy, intestinal metaplasia, and dysplasia. Based on pathology, three types of chronic gastritis are recognizable: [1] Diffuse antral gastritis which is usually due to *H. pylori* infection and could be accurately diagnosed through histology examination of a biopsy, [2] autoimmune metaplastic atrophic gastritis, and [3] metaplastic atrophic gastritis which develops in the course of chronic gastritis. During chronic inflammation of the stomach, the rate of cell loss may exceed the ability of stem cells to replace lost cells and mucosa thins. This phenomenon is often accompanied by metaplasia, and if associated with chronic inflammation is termed chronic atrophic gastritis or gastric atrophy.

Histologic diagnosis of atrophy is challenging; due to the lack of clarity in atrophy definition and severity (some consider metaplasia as an essential part of atrophy diagnosis) [12, 13], and its patchy nature (which requires a minimum of two biopsies from the incisura angularis and corpus), diagnostic accuracy of histology is imperfect in its specificity for atrophy. Other diagnostic methods such as serology share the same imprecision [14]. Therefore, in Scandinavian populations where gastric atrophy is not highly prevalent, the positive predictive value is low with a considerable number of false positive results. This non-differential misclassification of dichotomous exposure (atrophy vs. non-atrophy) forces the association toward null. It may explain the current study’s observation of a nonsignificant association between gastric atrophy and most of the specific causes of mortality which disagrees with the observed associations of metaplasia and gastritis with mortality risk.

Another challenging problem in the atrophy category is the admixture of its two major types with distinguished causes: autoimmune and *H. pylori*-induced atrophy. However clinical distinction between these two types is blurred, and a follow-up study of naive *H*. *pylori*-negative autoimmune atrophic gastritis cases did not show excess mortality from gastric cancer [15]. Yet, more studies are needed to understand whether the long-term behavior of the autoimmune type is distinct from *H. pylori*-induced atrophy.

Alternatively, atrophy can be considered as an adaptive response in which *H. pylori*-sensitive mucosa is replaced by the achlorhydric environment that is better able to withstand the hostile inflammatory stress. According to this argument, atrophy is a reversible mechanism that postpones the adverse effect of the extension of gastritis. Atrophy breaks the chain of persistent infection. It is observed that *H. pylori* do not colonize epithelium in a stomach that has undergone intestinal metaplastic changes following atrophy [16]. This hypothesis may partially explain the observation of no excess mortality from non-gastric malignancies in the non-metaplastic atrophy group. However, the change to atrophic mucosa comes with a price; although the epithelial lining is less acidic and less sensitive to *H. pylori*, mechanisms of protection against infection are lost, encouraging overgrowth of the stomach with non-*H. pylori* bacteria colonizing up- or down-stream mucosa (oral and intestinal microbiota). Moreover, the same influences that predispose mucosa to metaplasia, if persistent, may initiate malignant transformation in metaplastic atrophic cells. In this view, we expect that longer follow-up time would be required to observe excess mortality risk among non-metaplastic atrophy groups compared to metaplasia, and gastritis.

Association between gastritis, metaplasia, and dysplasia with elevated mortality due to respiratory diseases is of notable finding in this cohort. Respiratory diseases represent a leading cause of mortality and morbidity. Retrospective studies reported an inconsistent inverse association between *H. pylori* and asthma and a higher incidence of *H. pylori* seropositivity in chronic obstructive pulmonary disease (COPD) and chronic bronchitis. One of the potential confounders is socioeconomic status, which is associated with both *H. pylori* infection and the risk of developing chronic bronchitis. Smoking is another potential confounder, with a known higher incidence of COPD and lung cancer among smokers, yet variable infection rates of *H. pylori* have been observed among smokers. However, these confounding factors do not fully explain the observed associations in the aforementioned studies [17, 18]. Furthermore, previous studies have examined the potential role of *H. pylori* infection in the pathogenesis of respiratory diseases such as tuberculosis, cystic fibrosis, and sarcoidosis, yet contradictory results have been observed. Most of these studies relied on small-scale case–control studies and the assessment of exposure was based on seroprevalence, which significantly hampered to draw solid conclusions [17, 18]. The current study is by far the only large-scale prospective study that examined this association, revealing a potential indirect link between local consequences of *H. pylori* infection and respiratory diseases, meriting further investigation.

*H. pylori*-induced gastritis has been associated with protein-losing gastropathy and vitamin B12 deficiency. It may partly justify the observed elevated deaths due to endocrine and metabolic disease.

### Limitations of this study

The participants in our stomach biopsy cohort were not randomly sampled from the general population, but were those who underwent gastroscopy and biopsy due to specific indications. Our findings, thus, reflect a comparison of mortality risk among patients at varying Correa’s cascade stages within a clinically assessed group and are not directly applicable to healthy individuals without gastroscopy. Furthermore, precancerous conditions tend to manifest as patchy lesions, posing a certain risk of false-negative results. However, due to the prospective design and the 2-year latency, we essentially excluded most cases of existing gastric cancer and serious underlying diseases at baseline. Last, we lacked information on factors such as individual disease history, environmental exposures, and lifestyle factors, which could potentially aid in further risk stratification of patients.

## Conclusions

The findings in this unique cohort support the notion that the consequence of chronic inflammation of gastric mucosa is not limited to the stomach. Among extra-gastroduodenal organs and systems, excess mortality due to respiratory system disorders is of considerable notice and requires more investigation. Among types of diseases, malignancies (both gastric and non-gastric) were the most consistent disease types and their excess mortalities could be coherently linked to chronic inflammation in the stomach. For other organs and diseases either due to inconsistent results, a modest number of events, or the existence of other plausible explanations, we were unable to conclude a valid association.

### Supplementary Information

Additional file 1: Table S1. Systematized nomenclature of medicinediagnostic codes used in present cohort study. Table S2. Diagnosis codes used in mortality analysis

## Data Availability

Data are not publicly available due to their containing information that could compromise the privacy of research participants. Summary data can be made available from the corresponding author on reasonable request.

## Abbreviations

CI: Confidence interval
EGD: Esophago-gastro-duodenoscopy
HR: Hazard ratios
ICD: International Classification of Diseases
SMR: Standardized mortality ratio
SNOMED: Systematized Nomenclature of Medicine Morphology
