# Supplementary material for: Overall and cause-specific mortality among patients diagnosed with gastric precancerous lesions in Sweden between 1979 and 2014: an observational cohort study
Source: BMC Med. 2024 Aug 15;22:333. doi: 10.1186/s12916-024-03554-1 (PMC11328423; doi:10.1186/s12916-024-03554-1)
Supplement: Supplementary file 1 — Additional file 1: Table S1. Systematized nomenclature of medicinediagnostic codes used in present cohort study. Table S2. Diagnosis codes used in mortality analysis [file 12916_2024_3554_MOESM1_ESM.docx]

**Table S1.** Systematized nomenclature of medicine (SNOMED) diagnostic codes used in present cohort study

| SNOMED M code | Exposure group |
| --- | --- |
| **Correa’s cascade** |  |
| M01000 | Normal |
| Other M0 or M1/M2/M3XXXX† (not M38XXX) | Minor mucosal change |
| M4XXXX/ M38XXX | Gastritis (including gastric ulcer) |
| M58XXX | Atrophic gastritis |
| M73320 | Intestinal metaplasia |
| M74XXX | Dysplasia |

†X stands for any digits.

**Table S2.** Diagnosis codes used in mortality analysis

|  | ICD_7 | ICD_8 | ICD_9 | ICD_10 |
| --- | --- | --- | --- | --- |
| Cancer | 140-209 | 140-199 | 140-199 | C00-C97 |
| 'Gastric cancer' | 151 | 151 | 151 | C16 |
| 'All cancers other than GC' | 140-209 (except: 151) | 140-199 (except: 151) | 140-199 (except: 151) | C00-C97 (except: C16) |
| 'Lung cancer' | 162 | 162 | 162 | C34 |
| 'Infectious and parasitic diseases ' | 001-139 | 001-139 | 001-139 | A00-B99 |
| 'Endocrine, nutritional and metabolic diseases ' | 260-289 | 240-279 | 240-279 | E10-E90 |
| 'Mental and behavioral disorders' | 300-329 | 290-319 | 290-319 | F00-F99 |
| 'Nervous system' | 330-399 | 320-389 | 320-389 | G00-G99 |
| 'Circulatory system' | 400-469 | 390-459 | 390-459 | I00-I99 |
| 'Respiratory system' | 470-529 | 460-519 | 460-519 | J00-J99 |
| 'Digestive system' | 530-589 | 520-579 | 520-579 | K00-K99 |
| 'Genitourinary system' | 590-639 | 580-629 | 580-629 | N00-N99 |
| 'Pregnancy, childbirth and the puerperium' | 640-689 | 630-679 | 630-679 | O00-O99 |
| 'Symptoms, signs, and ill-defined conditions' | 780-799 | 780-799 | 780-799 | R00-R99 |
